# Speeding up Glioblastoma Cancer Research: Highlighting the Zebrafish Xenograft Model

**DOI:** 10.3390/ijms25105394

**Published:** 2024-05-15

**Authors:** Giusi Alberti, Maria Denise Amico, Celeste Caruso Bavisotto, Francesca Rappa, Antonella Marino Gammazza, Fabio Bucchieri, Francesco Cappello, Federica Scalia, Marta Anna Szychlinska

**Affiliations:** 1 Department of Biomedicine, Neurosciences and Advanced Diagnostics (BiND), University of Palermo, 90127 Palermo, Italy; giusi.alberti@unipa.it (G.A.); mariadenise.amico@unipa.it (M.D.A.); celeste.carusobavisotto@unipa.it (C.C.B.); francesca.rappa@unipa.it (F.R.); antonella.marinogammazza@unipa.it (A.M.G.); fabio.bucchieri@unipa.it (F.B.); francesco.cappello@unipa.it (F.C.); federica.scalia02@unipa.it (F.S.); 2 Euro-Mediterranean Institute of Science and Technology (IEMEST), 90139 Palermo, Italy; 3 The Institute of Translational Pharmacology, National Research Council of Italy (CNR), 90146 Palermo, Italy; 4 Department of Precision Medicine in Medical, Surgical and Critical Care (Me.Pre.C.C.), University of Palermo, 90127 Palermo, Italy

**Keywords:** glioblastoma multiforme, cancer, zebrafish, transgenic model, xenograft model, mammalian model

## Abstract

Glioblastoma multiforme (GBM) is a very aggressive and lethal primary brain cancer in adults. The multifaceted nature of GBM pathogenesis, rising from complex interactions between cells and the tumor microenvironment (TME), has posed great treatment challenges. Despite significant scientific efforts, the prognosis for GBM remains very poor, even after intensive treatment with surgery, radiation, and chemotherapy. Efficient GBM management still requires the invention of innovative treatment strategies. There is a strong necessity to complete cancer in vitro studies and in vivo studies to properly evaluate the mechanisms of tumor progression within the complex TME. In recent years, the animal models used to study GBM tumors have evolved, achieving highly invasive GBM models able to provide key information on the molecular mechanisms of GBM onset. At present, the most commonly used animal models in GBM research are represented by mammalian models, such as mouse and canine ones. However, the latter present several limitations, such as high cost and time-consuming management, making them inappropriate for large-scale anticancer drug evaluation. In recent years, the zebrafish (*Danio rerio*) model has emerged as a valuable tool for studying GBM. It has shown great promise in preclinical studies due to numerous advantages, such as its small size, its ability to generate a large cohort of genetically identical offspring, and its rapid development, permitting more time- and cost-effective management and high-throughput drug screening when compared to mammalian models. Moreover, due to its transparent nature in early developmental stages and genetic and anatomical similarities with humans, it allows for translatable brain cancer research and related genetic screening and drug discovery. For this reason, the aim of the present review is to highlight the potential of relevant transgenic and xenograft zebrafish models and to compare them to the traditionally used animal models in GBM research.

## 1. Introduction

Glioblastoma multiforme (GBM) is the most frequent and lethal primary brain cancer in adults. The annual incidence of GBM is 3–5 cases per 100,000 persons, with a slight prevalence in males compared to females [1,2]. Despite improvements in surgical and therapeutic fields in recent times, GBM continues to have a poor prognosis. GBM patients are an average of 64 years old at diagnosis [3], and the average survival rate is 14–15 months with standard treatment and 4–6 months without treatment. The survival rate 5 years after diagnosis is approximately 6% [4,5].

The multifactorial nature of GBM carcinogenesis, resulting from complex interactions between cells and the tumor microenvironment (TME), has posed great treatment challenges [6]. The standard treatment for GBM is surgical resection followed by chemo- and radiotherapy. However, the adaptive metabolic alterations, robust DNA repair, and self-renewing capabilities of glioma tumor cells promote resistance against the actual treatment approaches [7]. Enduring GBM management still requires the invention of innovative treatment strategies, and this has led to the necessity to develop in vitro and in vivo cancer models to properly evaluate the biological features and molecular mechanisms of tumor onset and progression. In recent years, the animal models used to study GBM tumors have undergone constant evolution, achieving highly invasive GBM models, which currently provide key information on the molecular mechanisms underlying the development of this tumor [8]. In vivo models are able to mimic important biological features of carcinogenesis, such as the TME, angiogenesis, immunological and inflammatory responses, and metabolic alterations, reflecting the heterogeneity and complexity of this tumor. Moreover, they offer valuable insights into the tumor’s molecular aspects, which are fundamental for evaluating potential therapeutic targets and drug discovery [8,9]. At present, the most commonly used animal models in GBM research are represented by mammalian models, such as mouse, canine, porcine, and other non-human ones, as well as non-mammalian models, such as the fruit fly, which have been widely used for their scope. However, traditionally used mammalian models present several limitations, such as high cost, more ethical issues, and time-consuming management, rendering them inappropriate for large-scale anticancer drug screening. In recent years, the zebrafish (*Danio rerio*) model has emerged as a valuable tool for studying GBM, and it has shown great promise in biomedical research and in preclinical GBM studies, allowing for translatable brain cancer research and related high-throughput genetic and drug screening [10,11].

For this reason, the aim of the present review is to highlight the potential of relevant transgenic and xenograft zebrafish models that have been investigated for GBM research and to compare them to the traditionally used animal ones, including a discussion of their advantages and limitations.

## 2. Multifaceted Aspects of GBM

**Figure 1:**
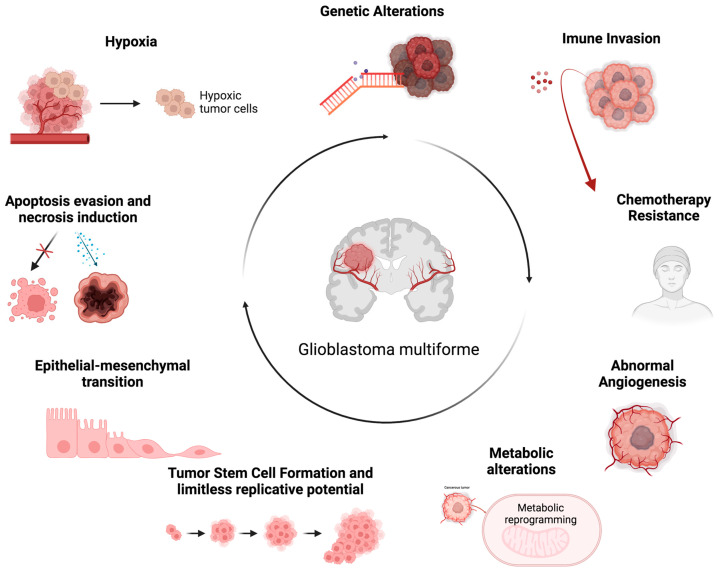
The representative scheme illustrating glioblastoma’s characteristic properties. The figure represents the hallmarks of GBM associated with multiple aspects of its pathogenesis and the consequent main causes associated with therapeutic resistance in GBM management. Created with Biorender.com, accessed on 14 May 2024.

GBM is a diffuse glioma that originates from astrocytes cells, and it can arise in any area of the central nervous system (CNS). However, they occur more frequently in the cerebral hemispheres [12]. The GBM cells present a polymorphic morphology with rounded or spindle-shaped cells, or very small or very large cells, with marked nuclear atypia and intense mitotic activity. Furthermore, an intense microvascular proliferation and large areas of necrosis are observed in the neoplasm area [12,13]. In the 2021 brain tumor classification, the World Health Organization (WHO), integrating histological features and molecular parameters, classified GBM as isocitrate dehydrogenase (IDH) wild-type and histone H3 wild-type diffuse glioma. GBM is characterized by several features, such as necrosis, microvascular proliferation, epidermal growth factor receptor (EGFR) gene amplification, mutation of the telomerase reverse transcriptase (TERT) promoter, and simultaneous gain of chromosome 7 and loss of chromosome 10 (+7/−10) [14,15]. GBM shows significant inter- and intra-tumoral heterogeneity, characterized by a complex TME particularly enriched in circulating tumor-infiltrating cells, such as glioma-associated macrophages (GAMs), tumor-infiltrating lymphocytes (TILs), and microglia. The latter represent approximately 25% of the tumor mass and play a key role in aberrant angiogenesis and in immunosuppression of the GBM microenvironment, two aspects that determine resistance to current therapies [16,17]. Furthermore, it is characterized by niches containing populations of GBM stem cells (GSCs) responsible for tumor spread, invasion, growth, and proliferation, further contributing to the development of therapy resistance [18,19,20]. The GBM microenvironment is a complex, dynamic, and interactive system. GBM cells and various components of the TME influence each other directly through cell–cell contact or indirectly through the release of soluble factors, such as cytokines, chemokines, growth factors, and extracellular matrix (ECM) remodeling enzymes, as well as via extracellular vesicles (EVs) [21,22,23,24,25,26]. In addition to these communication mechanisms, GBM cells use nanotubes, gap junctions, free DNA, horizontal DNA transfer, and circulating tumor cells to interact with other tumor and non-tumor cells. Overall, this complex communication network between GBM cells and TME components characterized by the release of altered metabolic products and chemical factors, such as pH and oxygen levels, promotes the proliferation, invasion, migration, and survival of GBM tumor cells [21,27,28,29]. Although GBM does not spread beyond nervous system structures, it represents a highly invasive tumor capable of rapidly spreading to surrounding tissues through the perivascular space of nearby blood vessels, through the space between neurons and neuroglia, or/and through the white matter fiber bundles. This property is attributable to several tumorigenic processes that develop in this context, such as ECM remodeling and epithelial–mesenchymal transition (EMT) [28,30]. The EMT is a critical mechanism in the invasiveness of GBM. The EMT causes separation of epithelia cells as they lose their basal–apical polarity and connection through tight junctions, taking on a fibroblast-like shape and increased mobility. The EMT concurrently determines the expression of mesenchymal biomarkers that promote the development of stemness features [31]. As the GBM cells progressively achieve the mesenchymal phenotype, they acquire greater migratory and tissue invasion capacity. GBM cells invade surrounding tissues undergoing numerous biological changes, resulting in cytoskeleton and ECM remodeling [32]. In this way, the ECM displays ligands, such as integrins, binding tumor cells and performing mesenchymal migration [33]. Another peculiar feature of GBM is a highly hypoxic TME [34]. Indeed, hypoxia represents a hallmark of GBM, and it is associated with multiple aspects of its pathogenesis (Figure 1) [6,30]. It is essential for the onset and progression of GBM, and it is an important pathogenetic factor related to tumor aggressiveness, its metastatic potential, and its therapy resistance [35]. In order to adapt to the hypoxic TME, the coordinated downregulation of metabolic demand to prevent bioenergetic collapse is required. The adaptive cellular response to hypoxia is mainly regulated through hypoxia-inducible factors (HIFs) [36]. In particular, hypoxia determines the rapid increase in HIF-1α expression within cells, which affects major signaling pathways. HIF-1α promotes glycolysis through the upregulation of glycolytic-pathway-critical enzymes, such as hexokinase 2 (HK2) and pyruvate dehydrogenase kinase 1 (PDK1). The upregulated expression of HIF-1α also affects GSCs, promoting their proliferation, infiltration, and self-renewal, as well as greater resistance to therapeutic irradiation [37]. Moreover, the downstream products of the HIF-1α signaling cascade include vascular endothelial growth factor (VEGF) [38], erythropoietin (EPO), and insulin-like growth factor 2 (IGF2), involved in tumor-induced angiogenesis. In a hypoxic environment, the GSC population persists and activates a series of adaptive mechanisms of neovascularization [39], including angiogenesis and vasculogenesis [40]. VEGF modulates angiogenesis in response to hypoxia, as well as promoting increased GSC survival. The GSC population is also involved in the mechanism of vasculogenesis, which leads to the formation of new blood vessels through the involvement of cells originating from the bone marrow [20]. Vascular proliferation is a histological hallmark of GBM. However, despite the apparent abundance of angiogenic activity, GBM is characterized by the malfunctioning of blood vessels [41]. The vessels are tortuous and permeable, lacking the structural support of the pericytes, and therefore preventing blood flow and resulting in lower perfusion. Frequent microvascular thrombosis is observed in tumor tissue, further occluding blood vessels and contributing to tissue hypoxia. In addition to the important role played in angiogenic activity, hypoxia helps to preserve the stemness of GSCs [42]. Its maintenance is promoted by Notch signaling activation [43]. HIF-1α physically interacts with the intracellular domain of Notch, stabilizing downstream signaling and preventing cellular differentiation of GSCs. Furthermore, constant hypoxia promotes the pathogenesis of GBM by promoting genomic instability, reducing the expression of DNA mismatch repair genes, and thus promoting the acquisition of new mutations. GBM presents complex metabolic alterations affecting several other cellular processes. Cancer cells have an increased requirement for nutrients for their rapid growth. Indeed, another hallmark of GBM cancer is the deregulation of cellular energy [44,45]. In this context, a key metabolic change is the Warburg effect [46], which is represented by an increase in glycolysis. GBM cells shift their metabolism towards using glycolysis rather than oxidative phosphorylation to generate ATP, even in the presence of oxygen. During glycolysis, in addition to energy generation, several intermediates are produced [47], promoting tumor growth and its progression. In addition, GBM cells display an upregulated pentose phosphate pathway (PPP) [48]. PPP is a metabolic pathway parallel to glycolysis that generates ribose-5-phosphate for nucleotide synthesis and NADPH for cellular redox balance. Upregulated PPP further supports the rapid growth and proliferation of GBM. GBM cells also show alterations in lipid metabolism [48]. Increased lipogenesis supports GBM growth by providing lipids for cell membrane synthesis and energy storage. In GBM, amino acid metabolism is also altered [49]. An increase in the uptake of glutamine is observed, which can be used as a carbon source for energy production and as a precursor for biosynthetic pathways. Moreover, an increase in glutamine absorption results in the stimulation of glutathione synthesis, which in turn helps fight against oxidative stress and is correlated with the increased capacity for radio- and chemotherapy resistance. Furthermore, the altered metabolism of arginine, a crucial component of the urea cycle, was shown to play a crucial role in tumor progression and immunosuppression [38]. GBM cells also produce higher levels of reactive oxygen species (ROS) [50], which contribute to tumor cell proliferation, survival, and invasiveness. They may also exhibit changes in mitochondrial function [51]. Mitochondria play a crucial role in energy production, the regulation of apoptosis, and several cellular metabolic processes. Mitochondrial DNA mutations can determine changes in the electron transport chain [52] and contribute to the establishment of an altered energy metabolism. In GBM, alterations in mitochondrial dynamics have been observed in relation to fission [53] and fusion, which contribute to the growth and proliferation of tumor cells. Moreover, GBM dysfunctional mitochondria increase ROS production [54], show alterations in membrane potential, and exhibit dysregulation in the mitochondrial apoptotic pathway [55], resulting in increased resistance to programmed apoptotic death and tumor cell survival [30].

## 3. Traditional Animal Models for In Vivo GBM Research

### 3.1. Mouse Models

The generation of GBM tumors in mice can be inducted through the use of chemicals or viruses but also through xenograft transplantation and somatic genetic modification [56]. Transplantation methods for rodent models of GBM can be accomplished in three ways: (i) injection of primary cancer cells into immunocompetent host mice; (ii) injection of human GBM cells into immunodeficient mice (patient-derived xenograft [PDX]); and (iii) implantation of genetically modified cells using oncogenes or tumor suppressor genes (genetically engineered model) [57,58]. The first experimental study describing the successful development of a glioma through intracranial (IC) implantation of 20-methylcholanthrene into C3H mice by Seligman and Shear [59] can be traced back to 1939, whereas in 1970 the first syngeneic GBM model was created and then maintained through the direct transfer of tumors [60] (Table 1).

The first viral induction dates back to the 1960s when the Rous sarcoma virus was used to induce GBM in mice; since then, considerable progress has been made in recent decades [58]. Currently, GBM models are no longer obtained using chemical compounds or viruses due to many challenges, including high maintenance requirements and incomplete tumor penetrance; however, several mouse cell lines obtained from such tumors or viruses engineered as transgenic gene vectors are widely used for mouse GBM models [65]. However, cell-line-based mouse models fail to reproduce all genetic, histological, and cellular features of the primary human tumor, resulting in poor translation into the clinic. More accurate preclinical mouse models are represented by PDX or genetically modified mouse models (GEMMs) capable of recapitulating different tumor properties and demonstrating sensitivity to drugs in vitro, with important implications in the clinical setting [66,67]. GBM GEMMs can be obtained through the use of a number of different methods, including conventional and conditional knockouts, transgenic mice, and many other viral techniques, and they are able to more closely reproduce the preclinical characteristics of this tumor, such as genetic and epigenetic alterations in tumor suppressor genes (Table 1) [61,62,63]. Some examples of models used in the study of GBM involve deletion of the tumor suppressor gene *p53*, which harbors a conditional allele of the tumor suppressor *Nf* [68]; the combined *Cdkn2a* knockout mouse models, with upregulation of *Kras* and *Akt* by viral induction [69]; the combined conditional knockout of the tumor suppressor genes *p53*, *Nf1*, and *Pten* [70]; and others. Recently, one methodology has also included using Cas9 regularly interspaced short palindromic repeats (CRISPR) technologies to create GBM GEMMs [71]. PDXs are widely used in preclinical studies for the validation of new therapeutic compounds and in the study of the genetic functions underlying the pathogenesis of GBM. Generally, human or mice glioma cell lines are injected into the subcutaneous flank location (heterotopic implantation) or into the brain (orthotopic implantation) of immunocompromised mice [72,73,74]. Because PDX models are obtained by implanting patient-derived cells into an immunocompromised mouse, they are able to maintain the histopathological, genomic, and phenotypic features of the primary tumor through the early stages (Table 1) [74]. Ponten et al. developed the best-known GBM xenograft model, i.e., the U251 glioma model, whose cells, once engrafted, are able to maintain the histopathological properties and the typical heterogeneity of this tumor, especially in the intracranial mouse model, compared to the subcutaneous model [75,76]. It was subsequently observed that injecting GSC lines into immunodeficient mice was able to induce a much more potent tumor capable of maintaining the typical heterogeneity of GBM, thus facilitating individual pharmacological screening and the search for resistance mechanisms [77]. Although these PDX models have shown favorable results in preclinical studies as they recapitulate many characteristics of their human counterpart, there are several factors that limit their usefulness, such as the following: (i) the orthotropic implant does not reproduce the conditions of the niche of origin of the tumor, thus limiting the study of the tumor’s biology and the mechanisms of resistance to therapy; (ii) these mouse models lack cells reactive to inflammatory processes or related to an intact immune system, therefore making them unsuitable for immunomodulatory therapies; and (iii) the development of important differences in selective pressures that occur during cell culture compared to the natural brain environment (Table 1) [64,74,77].

Overall, the advantages of mouse models are well-documented and contribute to their establishment as the dominant preclinical animal cancer model. However, there are key differences between mice and humans that contribute to their translational limitations, which include clear anatomical and physiological differences. As a result, there is a growing need for animal cancer models that can faithfully and reliably recapitulate human cancer.

### 3.2. Canine Models

Since the 1960s, canine models have been widely used in cancer research due to their high incidence rate of spontaneous tumor formation and their similarity to human tumors [78]. The canine model is phylogenetically closer to humans than its mouse counterpart. Among the types of brain tumors, meningiomas and gliomas are most frequent in dogs, with 43% and 32% incidence, respectively [79,80,81]. Considering GBM, it displays a similar histological pattern to human primary GBM, including GFAP/vimentin expression, necrosis, increased angiogenesis, hypercellularity, and inflammation. Similarly, compared to humans, the survival of dogs with GBM depends on the extent of resection, with greater survival for dogs undergoing total resection compared to those with subtotal resection, and they show a survival of a couple of months without treatment [82]. Furthermore, the infiltrative nature of spontaneous GBM in these models and the larger size of their brains make them interesting for testing new therapeutic treatments [83,84]. These models have also been used to study cell-mediated immunity against spontaneous GBMs [85]. In one of the first studies, stimulated autologous lymphocytes were implanted into the tumor environment in dogs following surgical removal and immunotherapy for recurrent malignant GBM, finding a decrease in tumor size [85]. In these canine GBM models, GSCs with important self-renewal and single-colony-formation abilities were also identified, and they expressed CD133 on their surface, a typical surface marker of GSCs reported in human gliomas. Of note, up-expression of EGFR, PDGFRα, and IGFBP2 has been identified in spontaneous canine glioma, which reflects the properties of the human counterpart [86,87]. The main advantage of studying spontaneous tumors in canine models is their spontaneous growth within a reasonable time frame, as well as the feasibility of treatment at various stages of the cancer process in a manner similar to human tumors [88]. Furthermore, in this animal model, spontaneous GBM presents an inter-patient heterogeneity that mimics that observed in human tumors, as different ages and dog breeds are able to develop tumors and GBM of different grades. Even intra-patient heterogeneity was found in spontaneous canine GBM characterized by polymorphonuclear cells, necrosis, vascular proliferation, and other characteristics typical of human GBM but absent in murine xenografts [89]. As a result, these animal models are becoming increasingly suitable for studying this brain tumor, in particular during the late stages of testing new therapies. However, canine GBMs are not as useful for the evaluation of single mutations underlying the origin of GBM, for which genetically modified mouse models are more suitable [87]. Another important limitation in the use of these canine models for experimentation is related to the practice of early euthanasia at different stages after the development of symptoms [90].

### 3.3. Porcine Models

A versatile large animal GBM model is the pig, which has a long history in biomedical research, with the sequencing of the genome of the domestic pig (*Sus scrofa*) completed in 2012 [91,92]. The pig brain is anatomically similar to the human cortex, and, for this reason, it can reproduce the administration and diffusion of drugs as well as tumor infiltration within these structures [93]. Other advantages include a larger brain size than humans, a high litter capacity (with up to 20 offspring each year), and, above all, fewer ethical limitations [94]. Currently, there are several useful porcine models for GBM studies obtained mainly after direct injection of human tumor cells into the brain with immunosuppression, as spontaneous tumor formation is rare in these models [95,96]. However, little is known about the characteristics of the porcine immune system and, therefore, its impact on xenograft models, other than the fact that serial cell cultures lead to the loss of genotypic and phenotypic heterogeneity in de novo glioma. The use of established tumor cells can also reduce the study of the events underlying tumor formation and progression. Finally, the large dimensions make the use of adequate equipment necessary, with considerable costs [97].

### 3.4. Non-Human Primate Models

Non-human primates (NHPs, such as *Rhesus* and *Cynomolgus macaques*) share broad similarities with humans physiologically, anatomically, and genetically, characteristics that better recapitulate tumor behavior and therapeutic responsiveness, providing an advantage in this direction [98,99]. Indeed, NHPs represent a particularly reliable experimental model species in which it is possible to study the etiology, pathogenesis, and response to treatment in brain diseases [100]. Yet, studies on NHP cancer models are limited to clinical cases and incidentally discovered tumor types, such as radiation-induced GBM in Macaca mulatta (*Rhesus macaque*), which is histologically and genomically similar to de novo human GBM, thus proving useful for therapy studies and pharmaceutical developments [101]. In addition to experimentally induced disease models, NHPs have long been employed as genetic models of diseases with key hereditary components for the study of cardiac disease [102], obesity [103], neurodegenerative disease [104], and hereditary cancers [105]. Although, for the above reasons, NHPs offer important advantages, cancer studies have faced significant limitations resulting from ethical concerns, extensive costs, and practical difficulties. Of notable importance is the lack of specific laboratory equipment essential for the management and genetic modification of large animal species, which limits the feasibility of the development of tumors in NHPs [106,107]. However, NHPs may constitute the transitional model species in research focused on humans to unravel the mechanisms of human diseases and to develop more in-depth therapeutic strategies.

### 3.5. Drosophila Melanogaster Model

Other animal models used to study GBM include the insect *Drosophila melanogaster* (fruit fly), which shares the same evolutionary mechanisms of neural development with humans, as well as 70% functional orthologous genes coding for proteins/components of important signaling pathways, such as oncogenesis (e.g., EGFR/RTK-Ras, PI3K, Jak-STAT, and TGF-β) [108,109,110,111]. Pioneering genetic studies in Drosophila are close to identifying that cancer may be linked to inherited genetic mutations, and that its genome contains numerous lethal mutants involved in the development of malignant diseases in various tissues, including the brain [112,113]. Moreover, the *Drosophila* nervous system has a very simple structure characterized by glial cells capable of performing functions similar to their vertebrate counterparts, including the mechanisms underlying reciprocal trophic exchange between neurons and glia, as well as glial cells capable of behaving as primary immune cells and therefore responding immunologically to neural trauma, and others [114]. For instance, the *Drosophila* model was used to create the Gal4-UAS system aimed at coactivating EGFR-Ras and PI3K in a glial-specific manner, thus allowing for the development of a tumor similar to human GBM starting from glial precursors [115]. The study has highlighted how the Drosophila model is able to give rise to gliomas starting from a few glial cells in a vascular context devoid of lymphocytes, exploiting mechanisms underlying the biology of human GBM that appear to be very well-conserved [116]. Additionally, neoplastic glial brain tumors have recently been developed in larval Drosophila in order to explore the pathogenic phenotypic effects of mutated genetic pathways and to identify potential therapeutic targets for tumors with these mutations [117]. The fly represents a model system that is easy to manipulate with current experimental techniques thanks to the fact that its genome is completely sequenced given its long history as an animal model in research [118]. Another advantage related to its usefulness in research is the short regeneration time (2–3 months) and no restrictions according to animal protection laws [119]. Despite these advantages, important species-specific differences must be considered that may not faithfully recapitulate the phenotypic and genetic characteristics of the human disease and related mouse models, including proliferation, invasion, as well as the evolution of altered metabolic pathways involved in the genesis of the GBM [120]. Furthermore, it is necessary to consider important aspects, such as the different anatomy of the fly brain from that of humans, the difficulties in using exclusively living cultures (no permanent conservation is possible), as well as the limited possibility of being able to study drugs, as their effects on the organism could differ greatly (such as the conversion of protoxins to toxins in the liver) [121,122].

## 4. Zebrafish (*Danio rerio*) Models in Cancer Research

Recently, zebrafish has emerged as an increasingly prominent non-mammalian vertebrate model in biomedical research. In particular, its use at both larval and adult developmental stages has gained great interest and rapidly expanded in neuroscience. Indeed, compared to traditional animal models, zebrafish have several advantages in scientific research. Firstly, female zebrafish is able to produce large cohorts of genetically identical offspring once a week. The eggs develop externally, permitting their easy manipulation. They require a low cost and ease husbandry due to their small size, permitting researchers to keep the embryos at different developmental stages within a limited space. Consequently, this enables significant statistical power of the studies and lower-cost experiments than mammalian models. Moreover, zebrafish develops rapidly and has a transparent nature during early developmental stages, allowing for real-time imaging and tracking of injected tumor cells at single-cell resolution. The embryo and larvae transparency enables researchers to observe and study the development of several organs and tissues through their real-time in vivo observation without invasive procedures [123]. The generation of transgenic lines with fluorescently labeled proteins of interest or fluorescent labeling of injected GBM cells permits the researchers to observe several tumor-associated dynamic processes, such as angiogenesis, TME interactions, and eventual immune responses. This technique also permits researchers to determine the effects of drugs and study the involvement of signaling pathways of interest in tumor progression. Additionally, complete sequencing of the zebrafish genome by the UK Sanger Institute demonstrated that zebrafish share a 70% homologous genome with humans, where approximately 80% of genes are human-disease-associated [124]. Moreover, zebrafish conserve several human anatomical features, such as the central nervous system (CNS) blood–brain barrier (BBB) and brain regions. This conservation of biological features suggests that the two species share some crucial biological pathways and makes zebrafish a valuable model for studying GBM and other cancers. Furthermore, zebrafish embryos are deprived of an adaptive immune system, eliminating the necessity for immunosuppression in xenograft experiments. Indeed, zebrafish lack adaptive immune system development until the first days post-fertilization (dpf), which renders this period ideal for the injection of tumor cells and the observation of their behavior within the fish organism [125]. Finally, the ease of executing microinjections to achieve genetic manipulations and to transplant cancer cells from patients enables researchers to recapitulate the biological features of human tumors and their related microenvironments, thus allowing them to further investigate both the tumor’s biological features and the mechanism of action of new therapeutic strategies [126]. However, the use of the zebrafish model for cancer research also presents some limitations. For instance, studies related to the pathways of INK4α/ARF, fundamental oncogenic factors, are not possible using this model as they are not expressed in zebrafish [127]. Moreover, some GBM-related epigenetic or metabolic alterations may represent a limitation in the zebrafish model. However, little is known in this regard, and it requires further investigation [128,129]. Another limitation of the model regards drug administration routes. The pharmacodynamic and pharmacokinetic process and interaction are different in zebrafish and humans, and they can further vary through the larval developmental stages. This poses significant impediments when it comes to drug efficacy or/and delivery studies, as results may be slightly different for humans [130]. However, several efficient routes for drug administration have been proposed and studied for zebrafish, including drug delivery systems to avoid uncontrolled delivery and to enhance therapeutic effectiveness [130]. Among them, the most employed techniques in zebrafish are represented by bath immersion, embryonic microinjection, oral administration, genetic manipulation, nanoparticles, and hydrogels, which have been extensively revised elsewhere [130].

### Transgenic and Transplantation (Xenograft) Zebrafish Models

**Figure 2:**
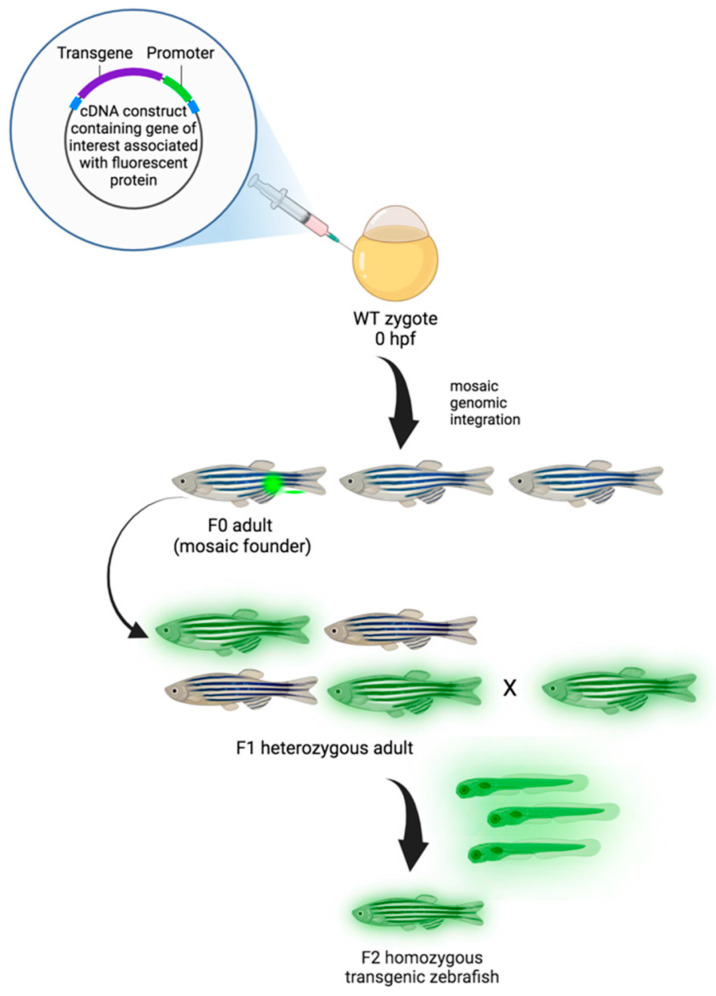
Generation of transgenic zebrafish model. Zebrafish embryos are injected with transgenic cDNA constructs at the one-cell stage and grown to maturity (F0). F0 adults are mosaic for the gene of interest conjugated with the fluorescent protein (transgene), and, when propagated, only those that underwent germline integration will produce offspring expressing the transgene in the F1 generation. Due to mosaicism within the germline, only a percentage of the F1 offspring may express the transgene. While the expression of transgenes is dominant, positive F1 animals are heterozygotes and need to be crossed to generate homozygous transgenic animals in the F2 generation [142]. Created with Biorender.com, accessed on 29 March 2024.

**Figure 3:**
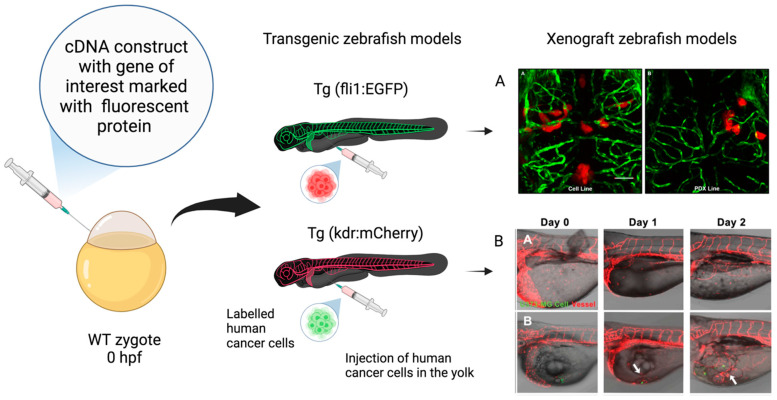
Xenograft zebrafish models in glioblastoma research. Schematic representation of transgenic zebrafish models and xenograft models used in GBM research. The transgenic fluorescent lines are created by embedding cDNA constructs containing the gene of interest associated with a fluorescent protein, such as enhanced green fluorescent protein (EGFP) and red fluorescent protein mCherry, at the one-cell-stage wild-type (WT) embryo. The xenograft model is obtained through the injection of labeled human glioma cells in the yolk sac at the 48–72 hpf stage. (**A**) Confocal images of zebrafish *Tg (fli1a:EGFP)^y1^* larvae brains injected with various GBM cell lines. (AA) Adult D54-MG-tdTomato cell line (red) and (AB) D2159MG pediatric xenoline (red). Adapted with permission from Umans et al., 2021 [133]. Copyright 2021, American Chemical Society. (**B**) Confocal images of zebrafish *Tg(kdr:mcherry)* larvae xenotransplantation model of U373-MG tumorsphere cells (green, white arrows). Adapted with permission from Lai et al., 2017 [138]. Copyright: © 2017 Lai et al. Created with Biorender.com, accessed on 29 March 2024.

In GBM research, the transgenic (Tg) zebrafish can be used for several experimental applications. Tg models are obtained through the embedding of DNA fragments into the zebrafish genome. This method permits the generation of transparent mutants (casper mutant strain) and/or achieving fluorescent protein stable overexpression in cells of interest (Figure 2). For instance, the Tg (fli1:EGFP) strain enables the investigation of vascular development by labeling friend leukemia integration 1 transcription factor (fli1), an early development endothelial cell marker, with enhanced green fluorescent protein (EGFP). This model has been largely utilized in GBM research to study neoangiogenesis and metastasis (see Figure 3) [131,132,133,134,135]. Similarly, zebrafish VEGF receptor kdr-like (Kdrl) has been employed to investigate blood vessel development by marking it with EGFP [135], green reef coral fluorescent protein (GRCFP) [136], or red fluorescent protein (mCherry) [137,138] (see Figure 3). Tg strains can also be used to investigate other cell types’ behavior and migration. For instance, macrophage and microglia visualization and tracking have been conducted by labeling macrophage-expressed gene 1 (*mpeg1*), an evolutionary conserved gene encoding perforin-2, in Tg (*mpeg1*:mCherry) [139] and Tg (*mpeg1*:EGFP) zebrafish strains. Moreover, the co-employment of *irf8*^−/−^ mutants lacking microglia can be useful in further investigating their the role in GBM progression and survival [140]. Zebrafish neural stem cells, astrocytes, and oligodendrocytes have also been defined by marking, respectively, the glial fibrillary acid protein (*gfap*) in Tg (*gfap*:GFP) [106] and oligodendrocyte transcription factor 2 (*olig2*) in Tg (*olig2*:GPF) [141] lines.

In Tg (*Huc*:GFP) [139,143], the fluorescence expression is limited to the neurons, as the HuC protein is designated as one of the earliest neuronal markers in zebrafish [144].

Furthermore, the remarkable potential of transgenic manipulation in zebrafish permits the development of strains that can be tailored to the precise research objective. For instance, the Tg (*βact*:Grx2) strain was used in a study by Wilms et al. to overexpress the oxidoreductase glutaredoxin 2 (*grx2*), which was associated with both wound healing cell migration and glioma cell invasion [141]. Furthermore, in a study by Rampazzo et al., the use of the Tg (*hsp70*:dkk-GFP) strain demonstrated that Wnt signaling can be temporarily suppressed by DKK1overexpression, thus determining the maintenance of the undifferentiated phenotype of injected GBM cells associated with higher mortality [145]. Finally, the Zebrabow strain has been employed to obtain in vivo multi-colored images [139,146].

In concert with transgenic models, zebrafish transplantation gained great attention as a useful model for biomedical research as well as for clinical studies when considering patient-derived xenografts (PDXs). This highly attractive model has been widely used in GBM research by several research teams because it faithfully reproduces tumor behavior in vivo [10,147]. The possibility to transplant patient cancer cells in zebrafish to evaluate and predict their aggressiveness, invasion, and metastatic potential characterizes this animal model as a valuable tool for prognosis. Compared to traditional rodent models, embryos of zebrafish require a minimal GBM cell number, permitting the generation of more heterografts from a single patient tumor [148]. Moreover, the possibility to label the grafted GBM cells is fundamental to track them within the animal body, thus providing information about GBM onset, infiltration, and progression and allowing for the investigation of innovative therapeutic strategies [149]. The transplanted GBM cells in zebrafish can be labeled using various techniques, such as luciferase enzyme reaction [137], fluorescent dyes (red, DsRed, CM-DiI, and green, DiO) [150], and optical reporter genes, such as red and green fluorescent proteins (RFP and GFP) [151]. Apart from cell labeling, there are several other critical parameters that have to be considered for successful xenotransplantation [2]. One of them is represented by the GBM cell line to be injected, which seems to play a crucial role in xenografting. The GBM cell lines and can be either laboratory- or patient-derived. Among the laboratory-derived cell lines, the malignant GBM G1261, C6, U87, and U251 cell lines are usually used, with U87 and U251 predominating [140,152]. Primary patient-derived GBM cells have also been commonly used, as they display higher tumor-initiating potential [135,153]. Moreover, different methods have been used for GBM cell line transplantation, including organoids, neurospheres [154,155], and attached cultures, with the latter displaying the most successful potential [135]. Other critical parameters are represented by the zebrafish’s developmental stage at grafting time as well as the injection site. Zebrafish embryos have very rapid development characterized by concomitant evolution of their anatomy, physiology, and immune system. GBM cell transplantation in embryonic stages performs better because the relative adaptive immune system has not fully developed at these stages and the procedure does not require immunosuppression. For instance, injection at the blastula stage (3.5–4.5 h post-fertilization, hpf) allows for rapid transplantation and does not necessitate sedation or precise orientation [156]. Nevertheless, the most widely used stage for tumor cell injection is at the beginning of the hatching period, corresponding to the pharyngula stage at 48 hpf. At this stage, zebrafish present a more defined brain morphology, along with vascular and lymphatic system development, supporting the tumor initiation and invasion and allowing researchers to study GBM biology in a more physiological-resembling microenvironment [157]. Moreover, xenotransplantation at 48 hpf offers a longer period (up to 5 days post-fertilization, dpf) for observations, presenting relatively less ethical/legislative issues compared to more advanced stages [158]. Several regions of developing zebrafish have been utilized as GBM cell injection sites at both embryonic and larval stages. Among the heterotopic injection sites, the blastoderm [139] and the yolk sac [159] at the blastula stage are the most frequently used, leading to the orthotopic intracranial tumor masses’ development. Orthotopic GBM cell xenotransplantation prevails in late larval stages, when the zebrafish brain’s microenvironment highly resembles the human one. In particular, the most commonly used injection sites during the larval stages are the ventricles or at the midbrain–hindbrain boundary, demonstrating a successful outcome [160]. Lastly, the temperature represents another fundamental parameter to be regulated when adopting a zebrafish GBM xenograft model. The optimal temperature between zebrafish and GBM cells has to be considered, resulting in the necessity to choose suboptimal temperatures that are tolerable by both species. The reported temperatures for the post-injection incubation condition range from 28 to 35 °C, with intermediate incubation between 31 and 33 °C [161]. Gradual adaptation of the xenografted zebrafish or development of a heat-tolerant Tg zebrafish model might improve GBM development [162]. In conclusion, as suggested in a systematic review by Pliakopanou et al. [11], the ideal zebrafish GBM xenograft model should be created using a standardized protocol to be employed in large-scale preclinical trials for anticancer drugs. In particular, the evidence from the literature suggest that Tg zebrafish strains orthotopically transplanted into the ventricle during the larval stage at 48 hpf with laboratory-derived cells (U87 cell line to study angiogenesis and U251 cell line to study GBM propagation) or PDX to accomplish clinical significance, gradually accustomed to 32–33 °C, could represent a reproducible and efficient zebrafish GBM model.

## 5. Comparative Analysis: Zebrafish vs. Traditional In Vivo Models for GBM Research

The existing preclinical GBM models are classified into two categories: xenografts and genetically engineered animal models. The xenografts obtained through the GBM cell-line injection have the advantages of high implantation and rapid growth rate, but it is undefined whether they mimic the tumor mass’s biological features. On the other hand, PDX is characterized by both histological and genetic aspects of the primary tumor, and, for this reason, it is expected to faithfully reproduce GBM biology. However, it cannot fully reproduce the anti-tumor immunological responses of the host [163,164]. Furthermore, genetically engineered GBM animal models permit the genetic manipulation involved in tumor onset and progression, but, normally, the tumors are formed by specific cells with homogeneous genetic alterations and are not able to mimic the intra-tumoral phenotypic and molecular heterogeneity. To unravel its complex biology and develop effective treatments, researchers rely on various in vivo models that mimic aspects of GBM pathogenesis [83,163,164]. Among these models, the zebrafish stands out for its unique advantages in developmental biology and genetic manipulation. However, several other established in vivo models, including mammalian (rodent and non-human primate) and non-mammalian (fruit fly) ones, offer distinct features and capabilities for GBM research [83,164]. However, there is still a need to establish innovative animal models that may fully recapitulate multifaceted human GBM biology [165]. The following table (Table 2) compares the zebrafish model with traditional mammalian models to provide researchers with a comprehensive understanding of their respective strengths and limitations in studying GBM. Finally, through an analysis of their genetic characteristics, tissue accessibility, technical considerations, life cycle dynamics, and resource demands, this comparison aims to assist researchers in selecting the most suitable model for their specific research objectives.

Common laboratory rodents (e.g., mice, rats) are small animals that have short generation times and are therefore highly available for use by various researchers. They present the BBB and consequently allow for the testing of drugs for neurological diseases. Despite this, these model systems present limitations related mainly to their genome, as only 85% of human genes have homologous orthologs in mice, and 20% of these perform very different functions (Table 2) [166]. Furthermore, human cells implanted in rodent xenograft models localize in a different microenvironment than human GBM and undergo different immune responses compared to those that develop in humans due to the impairment of the immune system in these animals [167]. As shown in Table 2, other limitations concern the need for careful control of variables dependent on genetic manipulations, breeding, and maintenance costs, as well as the time needed to address ethical issues. Considering these aspects and the fact that their phylogenetic relationship with NHPs is not so distant compared to other organisms used in the laboratory, such as zebrafish and Drosophila, they are the ones that most clearly delineate the limits as models for humans. Currently, models of non-primate mammals (such as the dog) are used in scientific research due to the advantages obtained from their use (Table 2). These model systems have larger organ sizes, more easily accessible tissues, and more human-like anatomy and physiology, and they are suitable for long-period follow-ups, as human patients are. For the same reason, it is also particularly difficult and expensive to breed and keep them in laboratories (Table 2) [102]. The greater genotypic, phenotypic, and physiological identity with humans compared to other model organisms belongs to NHPs, which therefore constitute an important resource for the study of human diseases. However, limitations inherent to the use of NHPs include cost, availability, ethical considerations, and, especially, longer time for experiments (Table 2) [113]. Zebrafish constitutes a promising model system for GBM xenotransplantation studies, although it is relatively new. There are several advantages acquired following the use of this model system, including the rapid development of embryos that are optically transparent, the high genomic homology with humans, which is essential for research and high-throughput pharmacological screening, and the ease of genetic manipulation (Table 2). However, despite being a valuable and promising animal model, Zebrafish also present several limitations, as stated above. One of them is represented by the optimal temperature difference between human cells and zebrafish embryos. Therefore, it is necessary to perform the experiments at a temperature between the optimal temperature for Zebrafish (i.e., 28.5 °C) and the xenografted GBM cells. Moreover, there are several limitations relative to the pharmacokinetics and pharmacodynamics, which may differ from those of humans. In addition, the GBM-related epigenetic and metabolic alterations may be subjected to differences and need to be further investigated. Ultimately, considering all of these aspects and depending on the specific aim of the study, once the protocol parameters, including the specific number of GBM cells, the specific zebrafish phenotype, the site of implantation, the timing of the injection, and the post-transplant maintenance temperature, are automated, the zebrafish model for GBM could become ideally perfect.

## 6. Future Prospects

Despite significant scientific efforts, the prognosis for GBM is still very poor, even after intensive treatment through surgery, radiation, and chemotherapy. To develop a novel therapeutic treatment for GBM, preclinical in vivo studies are essential for analyzing GBM’s biological features, evaluating new therapeutic targets, and assessing the potential of new therapeutic approaches. In recent years, in GBM research, much effort has been put into the study of the interactions between cancer and immune cells from the onset and along the development of the pathology. Moreover, several studies have discovered genetic, epigenetic, and metabolic changes observed in GBM, identifying these markers as potential targets for GBM diagnosis and treatment. In this context, xenograft zebrafish models have been successfully used to produce new insights into GBM pathology. In particular, with the advancement of precision medicine and light-relevant analytical techniques, the advantage of the transparent nature of zebrafish larvae permits the development of disease theranostics [165]. Overall, it is suggested that transgenic and xenograft zebrafish models should be further exploited for the evaluation of GBM-related biological process alterations and for the screening of new drugs, thus ultimately improving the diagnosis and treatment of this invasive tumor.

## Figures and Tables

**Table 1:** Comparative features of mouse GBM models.

Tumor Model	The Origin of the Tumor	Advantages	Disadvantages	References
Carcinogen-induced tumor model	Tumor induced by carcinogens	- Used to evaluate efficacy and toxicity of anticancer agents - Study of resistance and response biomarkers	- High animal mortality rate - Location and number of lesions are not uniform among individuals	[59]
Syngeneic tumor model	Transplanted mouse tumor cells	- Simple system able to recapitulate host immunity - Easily reproducible - Easy to manipulate	- It does not faithfully represent the tumor microenvironment - Reduced genetic heterogeneity of cells compared to the native tumor	[60]
Genetically engineered and viral-vector-mediated transduction model	De novo formed tumor induced by introduced mutations	- Models used to identify detailed information about the sequence of events underlying genetic alterations that occur in response to specific mutations - Adapted for the study of the microenvironment in tumor biology - Models suitable for preclinical and therapeutic studies	- Models often not representative of the genetic changes involved in GBM in humans - They do not faithfully reflect the intra-tumoral genetic and phenotypic heterogeneities of GBM therapeutic studies because of the beginning of the tumor reproducibility failure	[61,62,63]
Xenograft model of GBM (heterotopic)	Patient-derived tumor	- Models suitable for testing the effectiveness of drugs - Genetically stable	- An immunocompromised mouse is required to develop this model - It does not allow for testing of immunomodulatory therapies - It does not reproduce the original niche	[64]
Xenograft model of GBM (orthotopic)	Patient-derived tumor	- Models suitable for testing the effectiveness of drugs - Genetically stable - Models capable of maintaining the original tumor architecture and histological characteristics of the human tumor of origin	- An immunocompromised mouse is required to develop this model - It does not allow for testing of immunomodulatory therapies - It does not reproduce the original niche	[64]

**Table 2:** Comprehensive comparison of the zebrafish model with traditional rodent models and non-human primate models for studying GBM. This table highlights the distinct genetic characteristics, tissue accessibility, technical considerations, life cycle dynamics, and resource demands of each model, aiding researchers in making informed decisions regarding the selection of the most appropriate model for their GBM research endeavors.

Characteristics	Zebrafish Model	Rodent Models (e.g., Mice, Rats)	Non-Human Primate Models	Refs.
**Genetics and Manipulation**	Well-characterized genome, relatively simple genetic manipulation via CRISPR/Cas9.	Extensive genetic tools available, including transgenic and knockout technologies.	Closer genetic similarity to humans, enabling translational research but with higher technical demands.	[126,166]
**Size and Accessibility**	Small size, easy tissue observation and access for in vivo microscopy.	Larger size, variable accessibility depending on tumor location, and invasive procedures required.	Similar size to humans, facilitating surgical techniques and imaging studies, but with ethical and logistical challenges.	[95,124]
**Technical Drawbacks**	Lack of some genes conserved in humans.	Potential tumor heterogeneity due to different genetic backgrounds.	Ethical considerations, higher costs, and longer timelines for experiments.	[127,167]
**Life Cycle and Development**	Rapid life cycle and embryonic transparency facilitate tumor development studies.	Longer life span, enabling longitudinal studies and recapitulation of disease progression.	Longer life span, closer developmental timeline to humans, allowing for investigation of aging-related factors.	[95,123]
**Costs and Time**	Relatively low in terms of cost and time for model creation and maintenance.	Moderate costs for model creation and maintenance, varying depending on genetic manipulations.	Higher costs due to housing, care, and ethical considerations; longer timelines for experiments.	[106,123]
